# Supplementary material for: Assessment of the Nonlinear Electrophoretic Migration of Nanoparticles and Bacteriophages
Source: Micromachines (Basel). 2024 Mar 8;15(3):369. doi: 10.3390/mi15030369 (PMC10972334; doi:10.3390/mi15030369)
Supplement: Supplementary file 1 [file micromachines-15-00369-s001.zip › micromachines-2902275-supplementary.pdf]

micromachines

Supplementary Materials

Assessment of the Nonlinear Electrophoretic Migration of Nanoparticles and Bacteriophages

Adrian Lomeli-Martin [1], Zakia Azad [2], Julie A. Thomas [2,*] and Blanca H. Lapizco-Encinas [1,*]

[1] Microscale Bioseparations Laboratory, Biomedical Engineering Department, Rochester Institute of Technology, 160 Lomb Memorial Drive, Rochester, NY 14623, USA; al3577@rit.edu
[2] Thomas H. Gosnell School of Life Sciences, Rochester Institute of Technology, Rochester, NY 14623, USA; zsa2180@rit.edu
* Correspondence: jatsbi@rit.edu (J.A.T.); bhlbme@rit.edu (B.H.L.-E.)

Contents

LINEAR ELECTROPHORETIC VELOCITY MODEL FIT. **S2**

DIMENSIONLESS PARAMETERS EMPLOYED FOR IDENTIFYING THE APPROPRIATE NONLINEAR ELECTROPHORETIC REGIME: INCLUDES EQUATIONS S1 – S3 AND TABLES S1-S2 .... **S2**

PARTICLE AND PHAGE CONCENTRATION: INCLUDES TABLES S3 – S4 .... **S3**

INFORMATION ON PHAGE CHARACTERISTICS AND DIMENSIONS: INCLUDES TABLE S5 .... **S3**

CALCULATIONS OF THE MOBILITY OF NONLINEAR ELECTROPHORESIS AT THE ELECTROKINETIC EQUILIBRIUM CONDITION (E_{EEC}): INCLUDES EQUATION (S4) AND TABLE S6 .... **S3**

ESTIMATION OF THE HYDRODYNAMIC DIAMETER OF THE PHAGES: INCLUDES EQUATIONS S4-S7 .... **S4**

DETAILED DESCRIPTION OF CURRENT MONITORING EXPERIMENTS: **S4**

DETAILED DESCRIPTION OF PARTICLE TRACKING VELOCIMETRY (PTV) EXPERIMENTS: **S5**

ELECTROPHORETIC VELOCITY PLOTTED AGAINST ELECTRIC FIELD: INCLUDES FIGURE S1 .... **S5**

REFERENCES **S6**

Linear electrophoretic velocity model fit.

The well-known Helmholtz-Smoluchowski theory is only applicable when the particle radius (α) is much greater than the Debye length (λ_D), (i.e. $\kappa\alpha \gg 1$, where $\kappa = \lambda_D^{-1}$). The λ_D obtained for the suspending medium employed in this work was estimated to be 14.1 nm, which resulted in $\kappa\alpha$ values of 7.1-14.2 for particles and ~5.5 for phages, thus invalidating the use of the Helmholtz-Smoluchowski equation to describe linear EP migration in the current system. Under these conditions, where Debye length is comparable to the particle radius, Henry's equations should be applied. Henry's function, first proposed in 1933 by D.C. Henry [1], and later simplified by Ohshima in 1994 [2], is applicable for all values of $\kappa\alpha$ since it properly accounts for the electrophoretic retardation effect ignored by both the Helmholtz-Smoluchowski and Hückel models [2]. The simplified version of Henry's formula is shown in the main manuscript as Equation (2).

Dimensionless parameters employed for identifying the appropriate nonlinear electrophoretic regime: Includes Equations S1 – S3 and Tables S1-S2

The *Pe* number represents the ratio of convective to diffusive ion movement near the particle's surface. The *Du* number represents the ratio of surface conductivity (K^σ) to the bulk conductivity of the medium (K_m). Lastly, β corresponds to the ratio of the product between the applied electric field magnitude and α by the thermal voltage (φ), which is typically ~25 mV at 298 K. The expressions for these three dimensionless parameters are provided below [3]:

$$Pe = \frac{\alpha|\mathbf{v}_{EP}|}{D} \tag{S1}$$

$$Du = \frac{K^\sigma}{K_m \alpha} \tag{S2}$$

$$\beta = \frac{|\mathbf{E}|\alpha}{\varphi} \tag{S3}$$

where $\mathbf{v}_{EP}$ represents the EP velocity of the particle (both linear and nonlinear components), and D is the diffusion coefficient of the ions in the electrolyte solution.

Table S1. Weak field regime parameters, linear dependence ($\mathbf{E}^1$).

Particle or Phage ID	$\boldsymbol{\beta}$	$\boldsymbol{Du}$	$\boldsymbol{Pe}$	E for $\boldsymbol{\mu_{EP,L}}$ Estimation (V/cm)
Particle 1	0.013	1.707	0.004	50
Particle 2	0.026	0.751	0.010	50
Particle 3	0.026	0.959	0.010	50
SPN3US	0.019	1.322	0.009	50
ϕKZ	0.019	1.518	0.009	50

Table S2. Moderate field regime parameters, cubic dependence ($\mathbf{E}^3$).

Particle or Phage ID	$\boldsymbol{\beta}$	$\boldsymbol{Du}$	$\boldsymbol{Pe}$	E for $\boldsymbol{\mu^{(3)}_{EP,NL}}$ Estimation (V/cm)
Particle 1	0.414	1.707	0.251	1600
Particle 2	0.828	0.751	0.579	1600
Particle 3	0.828	0.959	0.487	1600
SPN3US	0.659	1.322	0.494	1700
ϕKZ	0.621	1.518	0.518	1500

Particle and phage concentration: Includes Tables S3 – S4

Shown below are the nanoparticle and phage concentration used in the PTV experiments. The information for the nanoparticles is shown summarized in **Table S1**, while the information for the phages is shown in **Table S2**.

Table S3. Nanoparticle concentration for PTV experiments.

Particle ID	*Particle Concentration (# of Particles mL^{-1})*
Particle 1	9.01×10^{10}
Particle 2	2.84×10^{10}
Particle 3	2.84×10^{10}

Table S4. Phage titers for PTV experiments.

Particle ID	*Particle titer (pfu mL^{-1})*
SPN3US	8×10^{12}
ϕKZ	8×10^{11}

Information on phage characteristics and dimensions: Includes Table S5

Information regarding the virion proteins, genome length, and estimates for the phage dimensions for both of the phages included in this study is presented in **Table S3**. The latter dimensions were used for the hydrodynamic diameter and sphericity calculations.

Table S5. Information on the number of virions proteins, genome length and dimensions of the phages employed in this study.

Phage ID	*No. of Different Virion proteins*	*Genome Length (bp)*	*Estimates of Virion Dimensions (nm)*	*Reference*
SPN3US	83	240,413	Capsid diameter: ~140<br>Tail length: ~200<br>Tail width: ~18	[4,5]
ϕKZ	62	280,334	Capsid diameter: 146<br>Tail length: 180<br>Tail width: ~20	[6,7]

Calculations of the mobility of nonlinear electrophoresis at the electrokinetic equilibrium condition (E$_{EEC}$): Includes Equation (S4) and Table S6

Shown below are the estimations of $\mu_{EP,NL}^{(3)}$ made at the electrokinetic equilibrium condition for the nanoparticles and phages included in this study. The values of $\mu_{EP,NL}^{(3)}$ at the E$_{EEC,}$ require the E$_{EEC}$ value to be interpolated from velocity data above and below the $\mathbf{v}_P = 0$ condition, employing the following expression as proposed by Cardenas-Benitez et al. [8]:

$$\mu_{EP,NL}^{(3)} = -\frac{(\mu_{EP,L} + \mu_{EO})}{E_{EEC}^2} \tag{S4}$$

As expected, these values in Table S6 are close to the values reported in Tables 1 and 2 of the main manuscript, which were estimated directly from the velocity data that was obtained at electric field that were the closest to the E$_{EEC}$ value.

Table S6. Values of $\boldsymbol{\mu_{EP,NL}^{(3)}}$ characterized at the EEEC.

Particle ID	$\boldsymbol{\mu_{EP,NL}^{(3)}}$ × 10-19 (m4V-3s-1)	EEEC (V cm−1)
Particle 1	−9.1 ± 2.1	1682.1 ± 373.7
Particle 2	−10.5 ± 2.5	1564.6 ± 379.8
Particle 3	−8.8 ± 2.0	1710.5 ± 389.0
SPN3US	−9.5 ± 2.7	1640.6 ± 463.1
ϕKZ	−12.5 ± 3.1	1434.0 ± 361.3

Estimation of the hydrodynamic diameter of the phages: Includes Equations S4-S7

For the calculations of the surface area and total volume of both phages, their shape was approximated into two shapes: an icosahedron (representing the protein capsid) and a cylinder (representing the tail). The radius of the circumscribed sphere around the icosahedron (i.e. the hydrodynamic radius of the capsid), is a parameter required for the surface area and total volume estimations. The following expression was utilized to estimate the hydrodynamics radius (r_H) of the icosahedron:

$$r_H = \frac{Capsid\ diameter}{2\ (0.8)} \tag{S5}$$

By considering the phage capsid as an icosahedron and the phage tail as a cylinder, the total surface area and total volume were obtained using the following expressions:

$$A_P = A_{cylinder} + A_{icosahedron} = \left[2\pi\ \frac{Tail\ width}{2}\ Tail\ length\right] + [8.7\ (r_H)^2] \tag{S6}$$

$$V_P = V_{cylinder} + V_{icosahedron} = \left[\pi\left(\frac{Tail\ width}{2}\right)^2\ Tail\ length\right] + [2.2\ (r_H)^3] \tag{S7}$$

The total surface area and total volume are required to estimate the sphericity, which is listed in Equation (7) in the main manuscript.

$$\psi = \frac{\pi^{\frac{1}{3}}(6V_P)^{\frac{2}{3}}}{A_P} \tag{7}$$

Finally, by employing the total volume, the hydrodynamic diameter (D_H) of the phages can be estimated by the following formula:

$$D_H = 2(\frac{3V_P}{4\pi})^{1/3} \tag{S8}$$

Detailed description of current monitoring experiments:

The following is a detailed description of the current monitoring experiments performed to measure the electroosmotic velocity in our system and is adapted from previous work from our group [9]. In steady EO flow, the total electric current flowing through a channel filled with the test solution (I_B) can be described considering contributions from the bulk solution and from the channel surface using the following equation:

$$I_B = A_C E_x \sigma_B^{bulk} + P E_x \sigma_B^{surface} \tag{S9}$$

where A_C and P are the cross-sectional area and perimeter of the channel, respectively, E_x is the x component of the electric field, σ_B^{bulk} is the conductivity of the bulk test solution, and $\sigma_B^{surface}$ is the channel surface conductance. The convection current has been neglected, since it is several orders of magnitude smaller than the currents presented in Equation (S9). If the time response of the electric current is linear, the EO velocity ($\mathbf{v}_{EO}$) can be directly calculated from the slope (m) of the current-time relationship.

$$\mathbf{v}_{EO} = \frac{m \cdot L}{(I_B - I_A)} \tag{S10}$$

where I_B and I_A are the current plateaus before and after the displacement process, respectively, and L is the length of the channel. As originally laid out by Almutairi et al. [10], both I_B and I_A are measured directly from the current-time data, since the current plateau values convey the experimental conductance of the bulk solution and channel walls. By combining Equation (1) from the manuscript and Equation (S10), the expressions for and are obtained:

$$\mathbf{v}_{EO} = \mu_{EO}\mathbf{E} = -\frac{\varepsilon_m \zeta_W}{\eta}\mathbf{E} \tag{1}$$

$$\mu_{EO} = -\frac{m \cdot L}{E_x \cdot (I_B - I_A)} \qquad \text{S11)}$$

$$\zeta_W = -\frac{m \cdot L \cdot \eta}{E_x \cdot \varepsilon_m \cdot (I_B - I_A)} \tag{S12}$$

Detailed description of particle tracking velocimetry (PTV) experiments:

For the PTV experiments, a series of DC voltages were programmed in the HVS6000D power supply sequencer and then applied between the electrodes at the inlet and outlet liquid reservoirs of the channel. Experiments consisted of one or two measurements done consecutively. First, a base voltage (V_B) of 25 V for 5 seconds to allow liquid flow to get started, followed by a target voltage (V_t) applied for 15 seconds at which the velocity measurements were taken. The base voltage was used to maintain the nanoparticles and phages in constant motion. ImageJ software was used for video analysis to estimate particle and cell velocities using particle image velocimetry (PIV). For all voltage applications, the x-axis velocity of 10 selected particles was tracked and averaged over the course of the 15 second voltage application. The complete assay was performed in triplicate per particle size using a new device and fresh media each time.

Electrophoretic velocity plotted against electric field: Includes Figure S1

Shown below is the electrophoretic velocity ($\mathbf{v}_{EP}$) of the nanoparticles (Figure S1a) and phages (Figure S1b) included in this study. This velocity was obtained by subtracting the electroosmotic component ($\mathbf{v}_{EO}$) to the overall particle velocity ($\mathbf{v}_P$). The $\mathbf{v}_{EP}$ values in Figure S1 include both linear and nonlinear electrophoresis ($\mathbf{v}_{EP} = \mathbf{v}_P - \mathbf{v}_{EO} = \mathbf{v}_{EP,L} + \mathbf{v}_{EP,NL}$).

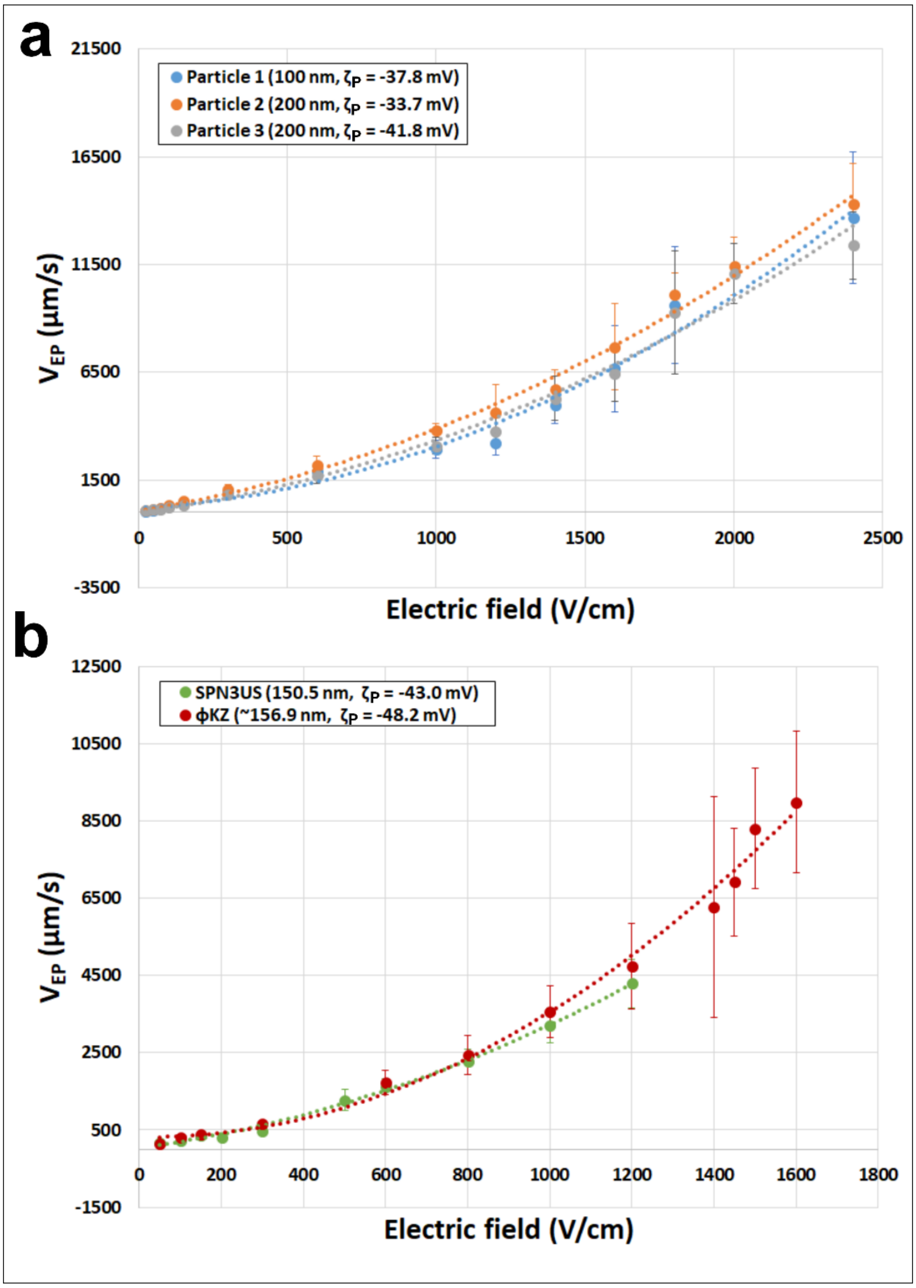

Figure S1. (**a**) Nanoparticle electrophoretic velocity ($\mathbf{v}_{EP}$, linear and nonlinear components) as a function of the electric field (E) (**b**) Phage electrophoretic velocity ($\mathbf{v}_{EP}$, linear and nonlinear components) as a function of the electric field (E). Each particle is represented in a different color, blue, orange, and grey for particle 1, particle 2, and particle 3, respectively, while SPN3US is shown in green and ϕKZ in red. Markers indicate experimental data and the dashed lines are included for ease of visualization. Error bars denote standard deviation.

References

1. Henry, D.C. The Cataphoresis of Suspended Particles. Part I. -The Equation of Cataphoresis. *Proceedings of the Royal Society of London. Series A* **1931**, *133*, 106–129, doi:10.1098/rspa.1931.0133.
2. Ohshima, H. A Simple Expression for Henry's Function for the Retardation Effect in Electrophoresis of Spherical Colloidal Particles. *J Colloid Interface Sci* **1994**, *168*, 269–271, doi:10.1006/jcis.1994.1419.
3. Schnitzer, O.; Yariv, E. Nonlinear Electrophoresis at Arbitrary Field Strengths: Small-Dukhin-Number Analysis. *Physics of Fluids* **2014**, *26*, 122002, doi:10.1063/1.4902331.
4. Scheuch, A.; Moran, S.A.M.; Faraone, J.N.; Unwin, S.R.; Vu, G.; Benítez, A.D.; Mohd Redzuan, N.H.; Molleur, D.; Pardo, S.; Weintraub, S.T.; et al. Mass Spectral Analyses of Salmonella Myovirus SPN3US Reveal Conserved and Divergent Themes in Proteolytic Maturation of Large Icosahedral Capsids. *Viruses* **2023**, *15*, doi:10.3390/V15030723/S1.

5. Bernard Heymann, J.; Wang, B.; Newcomb, W.W.; Wu, W.; Winkler, D.C.; Cheng, N.; Reilly, E.R.; Hsia, R.C.; Thomas, J.A.; Steven, A.C. The Mottled Capsid of the Salmonella Giant Phage SPN3US, a Likely Maturation Intermediate with a Novel Internal Shell. *Viruses* **2020**, *12*, doi:10.3390/V12090910.
6. Lecoutere, E.; Ceyssens, P.J.; Miroshnikov, K.A.; Mesyanzhinov, V. V.; Krylov, V.N.; Noben, J.P.; Robben, J.; Hertveldt, K.; Volckaert, G.; Lavigne, R. Identification and Comparative Analysis of the Structural Proteomes of ΦKZ and EL, Two Giant Pseudomonas Aeruginosa Bacteriophages. *Proteomics* **2009**, *9*, 3215–3219, doi:10.1002/pmic.200800727.
7. Fokine, A.; Battisti, A.J.; Bowman, V.D.; Efimov, A. V; Kurochkina, L.P.; Chipman, P.R.; Mesyanzhinov, V. V; Rossmann, M.G. Cryo-EM Study of the Pseudomonas Bacteriophage ΦKZ. *Structure* **2007**, *15*, 1099–1104, doi:10.1016/j.str.2007.07.008.
8. Cardenas-Benitez, B.; Jind, B.; Gallo-Villanueva, R.C.; Martinez-Chapa, S.O.; Lapizco-Encinas, B.H.; Perez-Gonzalez, V.H. Direct Current Electrokinetic Particle Trapping in Insulator-Based Microfluidics: Theory and Experiments. *Anal Chem* **2020**, *92*, 12871–12879, doi:10.1021/acs.analchem.0c01303.
9. Saucedo-Espinosa, M.A.; Lapizco-Encinas, B.H. Refinement of Current Monitoring Methodology for Electroosmotic Flow Assessment under Low Ionic Strength Conditions. *Biomicrofluidics* **2016**, *10*, 033104, doi:10.1063/1.4953183.
10. Almutairi, Z.A.; Glawdel, T.; Ren, C.L.; Johnson, D.A. A Y-Channel Design for Improving Zeta Potential and Surface Conductivity Measurements Using the Current Monitoring Method. *Microfluid Nanofluidics* **2009**, *6*, 241–251, doi:10.1007/s10404-008-0320-6.

Disclaimer/Publisher's Note: The statements, opinions and data contained in all publications are solely those of the individual author(s) and contributor(s) and not of MDPI and/or the editor(s). MDPI and/or the editor(s) disclaim responsibility for any injury to people or property resulting from any ideas, methods, instructions or products referred to in the content.
